# Optimal routing and end-to-end entanglement distribution in quantum networks

**DOI:** 10.1038/s41598-024-70114-1

**Published:** 2024-08-20

**Authors:** Joy Halder, Akhmadjon Rajabov, Riccardo Bassoli, Frank H. P. Fitzek, Gerhard P. Fettweis

**Affiliations:** 1 Vodafone Chair Mobile Communications Systems, Technische Universität Dresden, 01067 Dresden, Germany; 2 Deutsche Telekom Chair of Communication Networks, Technische Universität Dresden, 01067 Dresden, Germany; 3 Centre for Tactile Internet with Human-in-the-Loop (CeTI), Dresden, Germany; 4 Quantum Communication Networks (QCNets) Research Group, Technische Universität Dresden, Dresden, Germany

**Keywords:** Electrical and electronic engineering, Computer science, Information technology

## Abstract

Quantum networks are designed to transmit quantum bits (qubits) among quantum devices to enable new network resources for the applications. Entanglement distribution and entanglement swapping are fundamental procedures that are required in several network operations. However, they are probabilistic operations, which can lead to severe network performance degradation. This article investigates the engineering problem of resource allocation in quantum networks, considering factors like entanglement distribution probability, quantum memory characteristics, and fidelity. We model this as an optimization model to obtain an optimal solution. In particular, we formulate an integer linear programming (ILP) and develop a heuristic algorithm, aiming to minimize the number of required entangled qubit pairs (Bell pairs or EPR pairs) in any adjacent pair in the quantum network. Extensive simulations are performed to compare the performance of proposed ILP and heuristic. In all the cases, the heuristic produces a comparable solution to the optimal one. Simulation results ensure that the value of maximum utilized Bell pairs in a quantum network highly depends on the value of the probability of entangled pairs established, considering the time in the quantum memory besides the number of incoming requests.

## Introduction

Quantum communication networks have arisen significant attention since they can provide new fundamental resources to future communication networks. Several applications have started being investigated like information security^1^, communication^2^, quantum key distribution^3^, distributed quantum computing^4^, and quantum sensing^5^. These applications require a communication infrastructure that can distribute quantum bits (qubits) along with the classical bits^6^. Because of their different properties in respect of current communication networks, quantum networks cannot easily replicate the store-and-forward paradigm, which is currently widely-used: as qubits cannot be copied. This means that quantum networks require a major paradigm shift by adopting a set of new procedures to apply efficiently and effectively the laws of quantum mechanics at a network level.

A quantum network can formally be described as a distributed system, which connects different kinds of quantum devices (quantum nodes or Qnodes) to communicate, process, store, and sense quantum systems (e.g. qubits)^4,7,8^. Qubits can be classified into two categories: matter qubits and flying qubits^4,6^. The matter qubits are stored in quantum memories and are used by logic to interact via quantum gates to execute quantum algorithms and applications. Flying qubits can mainly be used to create entanglement among network nodes so that matter qubits at respective devices can interact. This can be realized by encoding the state of the qubit into the polarization of photons, transmitted via fiber optical links or free-space like in satellite links^9^. In quantum networks, entangled pairs can be distributed to establish a correlation that can be used by communication protocols, computing, and sensing applications. Using a Bell pair, a qubit can be teleported from one Qnode to another and when the teleportation completes, the Bell pair is consumed^10–12^. One of the main reasons to realise quantum networks is to reliably distribute entanglement in future networks so that applications can achieve performances that are not possible with classical technologies. In this context, routing plays a pivotal role for achieving optimal performances at application level. However, there exist several challenges in entanglement-based routing in a quantum network. First, qubits can interact with the environment by becoming noisy or losing its quantum properties. This phenomenon is known as decoherence, which exists and affects the process of qubit generation, qubit transmission, qubit storage, and qubit measurement. Second, qubits cannot be copied and stored, and hence cannot be transmitted to remote devices classically. Third, the creation of entanglement between two adjacent Qnodes is a probabilistic operation which may fail. Finally, life span of a successful entanglement creation is limited by quantum decoherence, i.e., each Bell pair is valid for a very short amount of time (typically, in order of a few seconds^9,13^. All these challenges must be taken into account while establishing a successful entanglement via routing protocols in a quantum network.

Entanglement distribution and entanglement swapping allow to couple multiple adjacent Qnodes. Hence, an end-to-end entanglement distribution can be achieved in between sources and destinations by performing entanglement swapping along a specific path. A quantum repeater performs entanglement swapping between two (flying) qubits, resulting in the end-to-end entanglement of two (matter) qubits^11,14^. Quantum repeaters are used in the intermediate Qnode(s) to perform the entanglement swapping operation without sending the matter qubits physically^10,15,16^. Hence, by inserting one or more than one repeater(s) between two Qnodes, the distance of entanglement distribution can be enhanced. However, like entanglement creation and distribution, entanglement swapping is a also probabilistic operation due to the imperfect swapping operation^12^. Moreover, the fidelity of Bell pairs is a real number in the range [0, 1], which indicates a relative deviation of a quantum state from the actual one^10,17^, where 1 denotes perfection. The fidelity of Bell pairs can degrade exponentially with the number of intermediate Qnodes, which further complexes the designing problem of routing in quantum networks. Entanglement purification is a potential solution to improve fidelity of a Bell pair^18–21^.

The problem of entangle-based routing and resource allocation in quantum networks growing its significance due to its vast application in several fields like quantum internet^7^, one-step quantum secure communication^22^, distributed quantum machine learning^23^, and so on. Several works consider the design of entanglement routing and the problem of resource allocation in repeater-based quantum networks. The entanglement-based routing problem in repeater quantum networks for a single flow is considered for the first time and quantum version of Dijkstra’s algorithm is adopted to solve this problem^14^. Later, several physical factors like decoherence time and entanglement success probability are considered on a variation of Dijkstra’s algorithm while design the routing metrics for quantum networks^24^. A time-slotted multipath-based approach is considered^25^ to enhance the rate of end-to-end entanglement distribution. In each time-slot, entanglement establishment is done in the adjacent Qnodes, next paths are determined based on successful entanglement establishment governed by a centralized processor. The routing protocol in a quantum network is further modified by adopting flawless entanglement swapping^26^. An integer linear programming (ILP)-based remote entanglement distribution model for homogeneous quantum repeater chain is developed^16^, without considering the properties of quantum memories. The design of entanglement distribution can be performed in two ways: pre-established and in real time^27^. Two heuristic algorithms are developed to solve the resource allocation problem in quantum networks, considering the design of entanglement distribution. A resource allocation model for distributed quantum computing, considering end-to-end entanglement distribution and entanglement success probability, is presented^17^. However, the optimal design of resource allocation in quantum networks, considering factors like entanglement distribution probability, the properties of quantum memories, fidelity for offline request set is still an open and unaddressed problem.

To address this unsolved problem, this article investigates the routing and end-to-end entanglement distribution problem for offline resource allocation on quantum networks. It proposes an optimization model to solve this problem, aiming to minimize the number of required Bell pairs in any adjacent Qnode pair in the quantum network. The optimization model is formulated as an ILP formulation to solve the problem optimally in small-sized quantum networks with a few number of requests. A heuristic algorithm is developed for large problem size, as the ILP formulation is tractable for small problem sizes only. In summary, the contributions of this work are described as follows.

1. We consider the routing and end-to-end entanglement distribution problem for offline resource allocation on quantum networks. Each request uses single path from respective source to destination nodes and end-to-end entanglement distribution is established along the selected path. We consider Bell pairs (EPR pairs) to establish entanglement between adjacent Qnode pairs. Fidelity of the selected paths must be greater than or equal to pre-determined threshold value. Each Qnode has a quantum memory consisting a set of memory cells with fixed memory time. All the memory cells update at the same time and facilitate by a centralized processor. Each request completes its execution within a single life-cycle of memory cells. Symmetric entanglement purification operation is performed for bit-flip channel model to improve the fidelity of Bell pairs.
2. We formulate an ILP formulation to solve the offline resource allocation problem in quantum networks considering end-to-end entanglement distribution. We develop a heuristic algorithm for the large problem size where ILP formulation becomes intractable.
3. We compare the performance of proposed ILP formulation and developed heuristic algorithm considering extensive simulation scenarios. Simulation results ensure that the developed heuristic provide near to optimal results obtained by the ILP formulation. The performance of the heuristic algorithm is then verified over a large quantum networks with higher number of requests considering different simulation setups.

The remainder of this paper is organized as follows. First, the background of this work is described by providing a brief review of routing and end-to-end entanglement distribution in quantum networks. Next, the proposed optimization model is formulated and the heuristic algorithm is developed. Finally, the experimental results of proposed ILP and heuristic are described.

## Background of routing and end-to-end entanglement distribution in quantum networks

In this section, the background of routing and end-to-end entanglement distribution in quantum networks is elaborately described and illustrated with an example on a sample quantum networks.

### End-to-end entanglement distribution model

The Bell pair-based entanglement establishment in two adjacent Qnodes of a quantum network involves following three operations: Bell pair generation in these Qnodes, entanglement swapping, and entanglement establishment^9,25,27^. At first, entangled qubit pair (Bell pair) is generated and distributed in the adjacent Qnode pair through quantum link. Next, quantum entanglement operation is performed between these Qnodes and the qubit pair is stored in memory cells of respective Qnodes. Finally, multiple entanglement swapping are performed in a chain of adjacent Qnodes to achieve an end-to-end entanglement distribution^27,28^.

The entanglement establishment operation using quantum repeaters is a probabilistic process and may fail due to quantum decoherence property and imperfect entanglement swapping^9,29^. If this operation fails, the qubits involved in the operation are discarded and a new entanglement establishment process begins with new set of entangled qubit pair. Let two adjacent Qnodes try to establish an entanglement operation with average measurement success probability *q*. If the demanded net rate is *B*, the number of Bell pairs required to successfully complete this operation is $Z = \lceil \frac{B}{q}\rceil$^11,16,17^.

The net rate *B* is the number of available Bell pair(s) available to quantum applications, and *Z* is the gross rate which denotes the number of Bell pair(s) consumed in those adjacent nodes of quantum network. In case of an end-to-end entanglement distribution involving *L* number of intermediate node(s), the end-to-end measurement success probability $Q_L$^17^ is given by $Q_L = q^L$. Hence, for a request *r* with net rate $B^r$ and $L^r$ number of intermediate node(s), the gross rate of request *r* $Z^r$ is given by Eq. (1)^17^:

1 $$ Z^{r}  = \left\lceil {{\text{ }}\frac{{B^{r} }}{{q^{L} }}} \right\rceil  $$

**Figure 1:**
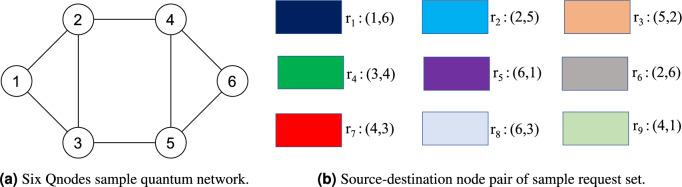
A sample quantum network and a set of requests to be placed on the quantum network.

The gross rate $Z^r$ denotes the number of required Bell pairs in each intermediate node in the routing path of request *r* to transmit $B^r$ qubit(s). In other words, the net rate is demanded by the request *r*, whereas the gross rate is that consumed from available network resources^17^. Table 1 reports the value of gross rate for different average measurement success probability *q* and *L* for a six nodes sample quantum network shown in Fig. 1a considering net rate *B*=1.

**Table 1:** Values of gross rate *Z* for different values of *q* using Eq. (1) on a six node quantum network for net rate *B*=1.

*q*	*L*				
	0	1	2	3	4
0.5	1	2	4	8	16
0.6	1	2	3	5	8
0.7	1	2	3	3	5
0.8	1	2	2	2	3
0.9	1	2	2	2	2
1.0	1	1	1	1	1

### Fidelity and entanglement purification

In quantum networks, fidelity of the Bell pairs drop whenever an entanglement swap is performed. The fidelity of Bell pairs degrade exponentially with the number of intermediate node(s) *L* and can be described by the Eq. (2) where $F_{\text {ini}}$ denotes the initial fidelity of Bell pairs^11,17^.

2 $$\begin{aligned} F = \frac{1}{4} + \frac{3}{4}\left( \frac{4F_{\text {ini}}-1}{3}\right) ^{L+1} \end{aligned}$$

From Eq. (2), following two cases can be concluded:

(i) if $F_{\text {ini}}$=1, then *F*=$F_{\text {ini}}$, i.e., no degradation in fidelity.
(ii) if $F_{\text {ini}}<$ 1, then *F* degrades exponentially with increased value of *L*.

In case of a quantum network with noisy intermediate scale quantum (NISQ) constraints, the value of $F_{\text {ini}}$ is less than 1, and hence *F* degrades exponentially according to case (ii). The value of *L* for a request depends on selected routing path based on several parameters, e.g., available number of Bell pairs in adjacent Qnodes, fidelity, and measurement success probability *q*^11,16,17^. Without fidelity constraint, for a quantum network with |*V*| number of Qnodes, range of *L* is $[0,|V|-2]$. However, if the resultant fidelity of a routing path must be greater than $F^{\prime }$, then $F^{\prime } \ge F$, and corresponding value of *L* from Eq. (2) is given by following equation^11^.

3 $$\begin{aligned} L+1 \le \left\lfloor \frac{\text {log} \left( \frac{4F-1}{3}\right) }{\text {log} \left( \frac{4F_{\text {ini}}-1}{3}\right) } \right\rfloor \end{aligned}$$

The fidelity of output Bell pairs depends on the fidelity of the two input states. With NISQ constraints after performing entangled distribution and quantum storage, the entangled states become mixed states due to several noise in the channel^18,20,30^. Entanglement purification becomes essential to extract maximally entangled states with high fidelity from mixed entangled states^18,20,21^. For example, in case of bit-flip channel model, entangled pair $\left| {\Psi ^+}\right\rangle$ may suffer a bit flip error and the corresponding mixed state can be represented using the following density operator $\varrho$^19,30^.

4 $$\begin{aligned} \varrho = F\left| {\Phi ^+}\right\rangle \left\langle {\Phi ^+}\right| + (1-F) \left| {\Psi ^+}\right\rangle \left\langle {\Psi ^+}\right| \end{aligned}$$

where $\left| {\Phi ^+}\right\rangle$ denotes a maximally entangled Bell pair.

In the entangled purification process for bit-flip channel model^18,20,21,31^, two CNOT operations are performed in between the pair of qubits of source and destination nodes at the same end of entanglement. Next, a measurement operation is performed on the qubits of second pair in a computational basis. If the qubit pairs are in the same state, the first pair is kept, otherwise it is discarded. This operation is successful if bit-flip error has occurred on both pairs or on no pairs^18,20,31^. The entanglement purification operation keeps the state with maximum fidelity from a pair of entangled states, and destroys the other state and its resources to the available network resources^18,20,31^.

If the input entangled pairs have fidelity $F_1$ and $F_2$, the probability of success and failure of the purification operation are $F_1 F_2 + (1-F_1)(1-F_2)$ and $F_1 + F_2 -2F_1 F_2$, respectively. The resultant fidelity after successful purification operation is given by Eq. (5)^18,30,31^.

5 $$\begin{aligned} F_{12} = \frac{F_1 F_2}{F_1 F_2 + (1-F_1)(1-F_2)} \end{aligned}$$

In case of symmetric purification operation, if both the input entangled pairs have same fidelity, i.e., $F_1$=$F_2$=*F* (say), then the resultant fidelity becomes $\frac{F^2}{F^2 + (1-F)^2}$^18^. The recursive computation of fidelity for $L+1$ swapping operation is given by Eq. (6)^18,30^, where $F_L$ is the fidelity of an entanglement state after performing purification operation in *L* intermediate node(s).

6 $$\begin{aligned} F_{L+1} = \frac{F^2_L}{F^2_L+ (1-F_L)^2} \end{aligned}$$

In this work, we assume symmetric purification operation for source Bell pairs and consider following two schemes to handle fidelity for routing and end-to-end entanglement distribution:

(i) without entanglement purification (w/o-P), where fidelity of a Bell pair is measured using Eq. (2)^11,17^,
(ii) with entanglement purification (w-P), where fidelity of a Bell pair is updated using Eq. (6)^18,30^.

### Entanglement-based routing in quantum network

The objective of routing problem in a quantum network is to find the best path for a given request in terms of given constraints such as flow constraints, available resources, minimum fidelity etc. $\gamma _{ij}$ denotes the capacity of adjacent Qnode pair (*i*, *j*), i.e., the rate at which Bell pairs are generated between (*i*, *j*)^17^. For a request $r \in R$, a routing path is selected in such a way that required number of Bell pairs (gross rate) along the path is minimum and fidelity constraint is satisfied. As both the fidelity and gross rate degrade exponentially with the number of intermediate node(s), selecting a routing path with a minimum number of intermediate node(s) is favourable. For example, if a request with *B*=1 selects a routing path with *L*=1, then gross rate $\gamma$ will be 2 for *q*=0.5. If the number of intermediate nodes become 3 (*L*=3), the gross rate $\gamma$ becomes 8 for the same value of *q*. The corresponding fidelity for *L*=1 and *L*=3 will be 0.9 and 0.82, respectively considering $F_{\text {ini}}$=0.95^11,17^.

### Bell pair generation window selection

In this work, we assume that each Qnode in a quantum network has a quantum memory and each quantum memory has a set of memory cells of fixed memory time $T_M$^25,27^. Adjacent Qnode pairs possesses entangled qubit pairs or Bell pairs and an entangled swapping operation consumes an entangled qubit pair. All the memory cells of the quantum network are updated simultaneously. The duration in between Bell pair (EPR pair) generation and termination is termed as a Bell generation window^25,27^. Each Bell generation window *w* has a fixed starting time $A_w$ and ending time $B_w$, i.e., all the Bell pairs are generated at time-stamp $A_w$ and terminated at time-stamp $B_w$ for each window *w*. The holding time of any request is much less than the quantum memory time $T_M$^27^. A request *r* with arrival time $a^r$, deadline $b^r$, and holding time $h^r$ can start at any time-stamp in the range $[a^r, b^r-h^r+1]$. However, each request must complete its execution within a single Bell generation window. Hence, a request *r* must select its starting time $\partial ^r$ from the range $[a^r, b^r-h^r+1]$ such that $\partial ^r \ge A_w$ and $\partial ^r + h^r -1 \le B_w$ for a Bell generation window *w*. Any request *r* must determine the set of Bell pair generation window(s) based on its arrival time, deadline, and holding time maintaining the mentioned constraints.

### System model

The quantum network is represented as a graph *G*(*V*, *E*) where *V* is the set of Qnodes, and *E* is the set of optical links. The quantum network consists of a centralized processor to govern end-to-end entanglement distribution between any pair of Qnodes. If there exist a link between two Qnodes *i* and *j*, Qnode pair (*i*, *j*) are referred as adjacent. *q* is the average measurement success probability of entanglement between any adjacent Qnode pair in *G*. In this work, we assume that the value of *q* is the same for all Qnodes in *G*^9^. Offline resource allocation model over a quantum network is considered where the request set *R* is known in advance. *R* is the set of requests to be placed in *G*, index *r*. $S^r$ and $D^r$ are the source and destination nodes of request *r*. $B^r$ denotes the demanded net rate of request $r \in R$. *L* denotes the set of possible number of intermediate node(s) of a routing path in *G*, index *l*. $a^r$ and $b^r$ are the arrival time and deadline of request *r*. $h^r$ is the holding time of request *r*. $T_M$ is the memory time of memory cells in each Qnode. It is assumed that all qubits in each Qnode have the same memory time^27^. *W* is the set of Bell pair generation windows, index *w*. $A_w$ and $B_w$ are the starting time of window *w*. Hence, in case of an Bell pair generation window *w*, all qubits in the quantum network *G* are generated at time $A_w$ and consumed at $B_w$. *T* is the set of time-stamps, index *t*. In other words, $|W|=\frac{T}{T_M}$. $U_{wt}$ is a constant, 1 if time-stamp *t* is in Bell pair generation window *w*, and 0 otherwise. $F^{\prime }$ is the maximum allowed fidelity of any routing path. *C* is a constant denotes the maximum number of intermediate nodes of any routing path so that fidelity constraint is satisfied according to Eq. (3).

The objective of this work is to determine a suitable routing path and select an Bell pair generation window and allocate resource for each request $r \in R$ in such a way that required number of Bell pairs in any adjacent node pair of the quantum network at any window is minimum.

### Illustration of proposed model

**Figure 2:**
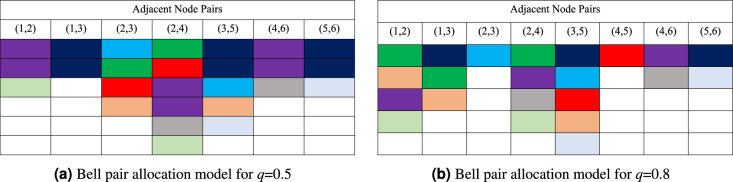
Snapshot of resource allocation model of request set over six-Qnodes sample quantum network for *q*=0.5 and *q*=0.8.

The proposed resource allocation model is illustrated using Fig. 1. A six-Qnodes sample quantum network is shown in Fig. 1a. Nine requests are considered with random source-destination Qnode pair shown in Fig. 1b. The net rate of each request (*B*) is set to 2 and only one Bell pair generation window is considered so that all requests can be placed in the same window. A snapshot of resource allocation of the request set for *q*=0.5 and *q*=0.8 are shown in Figs. 2a,b, respectively. In Fig. 2, each rectangular block represents four Bell pairs distributed in adjacent Qnode pair. Fig. 2 plots end-to-end Bell pair distribution along selected routing path for each request, and plot resource allocation model by showing the consumed Bell pairs in each adjacent Qnode pair. The fidelity constraint is not considered in this example to allow requests adopt routing paths of any length.

In case of resource allocation model for *q*=0.5 (Fig. 2a), each request uses routing path with minimum number of intermediate node(s). For example, source-destination node pair of request $r_3$ are (5,2) and the shortest paths in terms of number of intermediate nodes are 5-3-2 and 5-4-2 respectively, on which path 5-3-2 is selected. The gross rate of $r_3$ is 4 as it has one intermediate node (see Table 1), that is 4 Bell pairs are used in each of the node pairs (5,3) and (3,2). In this resource allocation model, the maximum number of Bell pairs used in the quantum network is 24 for adjacent node pair (2,4).

In case of resource allocation model for *q*=0.8 (Fig. 2b), the maximum number of Bell pairs consumed in the quantum network is 20 for adjacent node pair (3,5). However, requests $r_3$ and $r_4$ selected routing paths have higher number of intermediate nodes compared to the shortest paths, e.g., $r_3$ uses path 5-3-1-2 with two intermediate Qnodes. The gross rate of $r_3$ is 4 (see Table 1), that is 4 Bell pairs are consumed in each of the node pairs (5,3), (3,1), and (3,2). The same is true for $r_4$ with routing path 3-1-2-4. It is evident that with an increase in the value of *q*, the gross rate decreases and requests are allowed to use longer routing paths, provided the fidelity constraint is satisfied.

## Proposed optimization model

The proposed resource allocation model to optimize the number of consumed Bell pairs due to end-to-end entanglement distribution is formulated as an ILP problem. The objective of this ILP formulation is to minimize the maximum number of Bell pairs required in between any adjacent Qnode pair. The set of constants, variables, and constraints are described as follows.

The constants used in this ILP formulation are: $\triangle$ is a positive constant sufficiently large than total net rate by all requests, and $\varepsilon$ is a small positive constant such that $0< \varepsilon <1$.

The variables used in this ILP formulation are described below. $x^r_{ij}$ is a binary variable, 1 if routing path of request *r* uses link (*i*, *j*), and 0 otherwise. $y^r_i$ is a binary variable, 1 if *i* is an intermediate node in routing path of request *r*, and 0 otherwise. $z^r$ is an integer variable denoting the number of intermediate node(s) in routing path of request *r*. $\alpha ^r_l$ is a binary variable, 1 if routing path of request *r* has *l* intermediate node(s), and 0 otherwise. ${\overline{\rho }}^r$ is an integer variable denoting the gross rate of request *r*. $\beta ^r_{ij}$ is an integer variable denoting the required number of Bell pair(s) in the adjacent node pair (*i*, *j*) for request *r*. $\phi ^r$ is an integer variable denoting the starting time of request *r*. $\omega ^r_w$ is a binary variable, 1 if request *r* selects window *w*, and 0 otherwise. $\partial ^r$ is an integer variable denoting the starting time of selected window by request *r*. In other words, $\partial ^r = A_w$ if $\omega ^r_w = 1$. ${\overline{\partial }}^r$ is an integer variable denoting the ending time of selected window by request *r*. In other words, ${\overline{\partial }}^r = B_w$ if $\omega ^r_w = 1$. $\psi ^r_t$ is a binary variable, 1 if $t \ge \phi ^r$, and 0 otherwise. ${\overline{\psi }}^r_t$ is a binary variable, 1 if $t \le \phi ^r + h^r -1$, and 0 otherwise. $\Psi ^r_t$ is a binary variable, 1 if $t \ge \phi ^r$ and $t \le \phi ^r + h^r -1$ simultaneously, and 0 otherwise. In other words, $\Psi ^r_t =1$ if *r* is active in *G* at time-stamp *t*, and 0 otherwise. $Y^r_{ijt}$ is a binary variable, 1 if request *r* uses node pair (*i*, *j*) at time-stamp *t*, and 0 otherwise. $Z^r_{ijt}$ is an integer variable denoting the number of Bell pair(s) required in the adjacent node pair (*i*, *j*) for request *r* at time-stamp *t*. $\pi ^r_{ijw}$ is an integer variable denoting the total number of Bell pair(s) required in the adjacent node pair (*i*, *j*) at window *w*. In other words, $\pi ^r_{ijw}$=$Z^r_{ijt}$ if values of $\omega ^r_w$ and $U_{wt}$ are 1 simultaneously. $\gamma _{ijw}$ is an integer variable denoting the total number of Bell pair(s) required in the adjacent node pair (*i*, *j*) at window *w*. $\Gamma$ is an integer variable denoting the maximum number of Bell pair(s) required in any adjacent node pair in *G*.

The ILP formulation is described as follows.

7 $$\begin{aligned} \textrm{Minimize} \quad \Gamma \end{aligned}$$

Subject to the following constraints.

$\bullet$ Routing path selection constraints.

8a $$\begin{aligned}{} & {} \sum _{j:(i, j)\in E} x^r_{ij} -\sum _{j:(j, i)\in E} x^r_{ji} = \left\{ \begin{array}{l l l} 1 &{} \text{ if } \quad i = S^r,\\ -1 &{} \text{ if } \quad i = D^r, \quad \forall r, i \\ 0 &{} \text{ otherwise. } \end{array} \right. \end{aligned}$$

8b $$\begin{aligned}{} & {} \sum _{j \in V} x^r_{ij} = y^r_i, \quad \forall r, i: i \ne S^r, D^r \end{aligned}$$

8c $$\begin{aligned}{} & {} \sum _{i \in V} y^r_i = z^r, \quad \forall r \end{aligned}$$

8d $$\begin{aligned}{} & {} z^r \le C, \quad \forall r \end{aligned}$$

Equation (8a) determines the routing path of each request *r* using flow-constraints on node *i* for each request *r*. If *i* is an intermediate node in routing path of request *r*, (8b) sets the value of $y^r_i$ as 1, and Eq. (8c) computes the total number of intermediate node(s) in the routing path of request *r*. Eq. (8d) ensures the maximum number of intermediate node(s) in any path must be less than or equal to *C* to ensure fidelity constraint.

$\bullet$ Gross rate computation constraints.

9a $$\begin{aligned}{} & {} \triangle (1 - \alpha ^r_l) \ge z^r - l, \quad \forall r, l \end{aligned}$$

9b $$\begin{aligned}{} & {} \sum _{l \in L} \alpha ^r_l = 1, \quad \forall r \end{aligned}$$

9c $$\begin{aligned}{} & {} \rho ^r = \sum _{l \in L} \alpha ^r_l Q_l, \quad \forall r \end{aligned}$$

Equations (9a) and (9b) set the value of $\alpha ^r_l$ as follows. If $z^r > l$, then right-hand side of (9a) becomes positive which ensures $\alpha ^r_l$=0. When $z^r \le l$, (9a), then left-hand side of (9a) becomes negative which ensures that $\alpha ^r_l$=1 so that right-hand side of (9a) equals to 0. Hence, value of $\alpha ^r_l$ will be 1 only when $l \ge z^r$. Finally, (9b) ensures that for any request *r*, $\alpha ^r_l$ will be 1 for only one value of *l* from the set of allowable value(s). Eq. (9c) computes the gross rate for request *r* based on selected *l* value.

$\bullet$ Bell pair pair allocation constraints.

10a $$\begin{aligned}{} & {} \beta ^r_{ij} \le \triangle x^r_{ji}, \quad \forall r, i, j \end{aligned}$$

10b $$\begin{aligned}{} & {} \beta ^r_{ij} \ge B^r {\overline{\rho }}^r - \triangle (1- x^r_{ji}), \quad \forall r, i, j \end{aligned}$$

Equations (10a) and (10b) define $\beta ^r_{ij}$ as follows. If request *r* does not uses l  (*i*, *j*) (i.e., $x^r_{ji}$=0), (10a) sets $\beta ^r_{ij}$=0, otherwise some positive value less than or equal to $\triangle$. If $x^r_{ij}$=1, (10b) sets the value of $\beta ^r_{ij}$ to $B^r {\overline{\rho }}^r$ as $\triangle (1- x^r_{ji})$ becomes 0.

$\bullet$ Bell pair generation window selection and starting time allocation constraints.

11a $$\begin{aligned}{} & {} \sum _{w \in W} \omega ^r_w = 1, \quad \forall r \end{aligned}$$

11b $$\begin{aligned}{} & {} \partial ^r = \sum _{w \in W} \omega ^r_w A_w, \quad \forall r \end{aligned}$$

11c $$\begin{aligned}{} & {} {\overline{\partial }}^r = \sum _{w \in W} \omega ^r_w B_w, \quad \forall r \end{aligned}$$

11d $$\begin{aligned}{} & {} \phi ^r \ge a^r, \quad \forall r \end{aligned}$$

11e $$\begin{aligned}{} & {} \phi ^r \ge \partial ^r, \quad \forall r \end{aligned}$$

11f $$\begin{aligned}{} & {} \phi ^r + h^r -1 \le b^r, \quad \forall r \end{aligned}$$

11g $$\begin{aligned}{} & {} \phi ^r + h^r -1 \le {\overline{\partial }}^r, \quad \forall r \end{aligned}$$

Equation (11a) states that each request selects one and only one window for its execution. Equations. (11b) and (11c) computes starting and ending time of the window selected by request *r* based on $\omega ^r_w$ by (11a). Equations (11d) and (11e) ensure that the starting time of request *r* must be greater than or equal to both the starting time of the selected window ($\partial ^r$) and arrival time of request *r* ($a^r$). Equations (11f) and (11g) ensure that request *r* completes its execution before the ending time of the selected window (${\overline{\partial }}^r$) and deadline of request *r* ($b^r$).

$\bullet$ Execution time computation constraints.

12a $$\begin{aligned}{} & {} \triangle \psi ^r_t \ge t - \phi ^r + \varepsilon , \quad \forall r, t \end{aligned}$$

12b $$\begin{aligned}{} & {} \triangle (1-\psi ^r_t) \ge \phi ^r -t, \quad \forall r, t \end{aligned}$$

12c $$\begin{aligned}{} & {} \triangle {\overline{\psi }}^r_t \ge (\phi ^r + h^r -1) - t + \varepsilon , \quad \forall r, t \end{aligned}$$

12d $$\begin{aligned}{} & {} \triangle (1-{\overline{\psi }}^r_t) \ge t - (\phi ^r + h^r -1), \quad \forall r, t \end{aligned}$$

12e $$\begin{aligned}{} & {} \Psi ^r_t \le \psi ^r_t, \quad \forall r, t \end{aligned}$$

12f $$\begin{aligned}{} & {} \Psi ^r_t \le {\overline{\psi }}^r_t, \quad \forall r, t \end{aligned}$$

12g $$\begin{aligned}{} & {} \Psi ^r_t \ge \psi ^r_t + {\overline{\psi }}^r_t -1, \quad \forall r, t \end{aligned}$$

Equations (12a) and (12b) define $\psi ^r_t$ as follows. If *t* is greater than or equal to $\phi ^r + \varepsilon$, right-hand side of (12a) becomes positive, and hence (12a) forces to set $\psi ^r_t$=1. In a similar way, if *t* is less than $\phi ^r + \varepsilon$, (12a) forces to set $\psi ^r_t$=0. Equations (12c) and (12d) define ${\overline{\psi }}^r_t$ in the same way as (12a) and (12b) define $\psi ^r_t$. Equations (12e), (12f), and (12g) perform logical ‘AND’ operation of $\psi ^r_t$ and ${\overline{\psi }}^r_t$ to determine $\Psi ^r_t$ as follows. If any of $\psi ^r_t$ and ${\overline{\psi }}^r_t$ is 0, (12e) and (12f) force to set $\Psi ^r_t$=0. If both $\psi ^r_t$ and ${\overline{\psi }}^r_t$ are 1, (12g) forces to set $\Psi ^r_t$=1. Hence, $\Psi ^r_t$ is set to 1 only when both $\psi ^r_t$ and ${\overline{\psi }}^r_t$ are 1.

$\bullet$ Execution time allocation constraints.

13a $$\begin{aligned}{} & {} Y^r_{ijt} \le x^r_{ij}, \quad \forall r, t \end{aligned}$$

13b $$\begin{aligned}{} & {} Y^r_{ijt} \le \Psi ^r_t, \quad \forall r, t \end{aligned}$$

13c $$\begin{aligned}{} & {} Y^r_{ijt} \ge x^r_{ij} + \Psi ^r_t -1, \quad \forall r, t \end{aligned}$$

Equations (13a), (13b), and (13c) perform logical ‘AND’ operation of $x^r_{ij}$ and $\Psi ^r_t$ to determine $Y^r_{ijt}$. If any of the $x^r_{ij}$ and $\Psi ^r_t$ is 0, (13a) and (13b) set $Y^r_{ijt}$=0. If both $x^r_{ij}$ and $\Psi ^r_t$ are 1, (13c) ensures $Y^r_{ijt}$=1.

$\bullet$ Objective function definition constraints.

14a $$\begin{aligned}{} & {} Z^r_{ijt} \ge \beta ^r_{ij} - \triangle (1-Y^r_{ijt}), \quad \forall r, i, j, t \end{aligned}$$

14b $$\begin{aligned}{} & {} \pi ^r_{ijw} \le \triangle \omega ^r_w U_{wt}, \quad \forall r, i, j, w, t \end{aligned}$$

14c $$\begin{aligned}{} & {} \pi ^r_{ijw} \ge Z^r_{ijt} - \triangle (1 - \omega ^r_w U_{wt}), \quad \forall r, i, j, w, t \end{aligned}$$

14d $$\begin{aligned}{} & {} \gamma _{ijw} = \sum _{r \in R} \pi ^r_{ijw}, \quad \forall r, i, j, w \end{aligned}$$

14e $$\begin{aligned}{} & {} \Gamma \ge \gamma _{ijw} + \gamma _{jiw}, \quad \forall i, j, t \end{aligned}$$

Equation (14a) defines $Z^r_{ijt}$ as follows. If $Y^r_{ijt}$=1, $\triangle (1-Y^r_{ijt})$ becomes 0 and (14a) assigns $\beta ^r_{ij}$ to $Z^r_{ijt}$, otherwise $Z^r_{ijt}$ is set to 0. Equations (14b, 14c) define $\pi ^r_{ijw}$ as follows. If both $\omega ^r_w$ and $U_{wt}$ are 1, only then (14b) assigns some positive value to $\pi ^r_{ijw}$; and 0 otherwise. If $\pi ^r_{ijw}$ is not set to 0, (14c) sets $\pi ^r_{ijw}=Z^r_{ijt}$. Note that (14b) and (14c) perform ‘AND’ operation of integer variable $Z^r_{ijt}$, binary variable $\omega ^r_w$, and constant $U_{wt}$ to obtain the integer variable $\pi ^r_{ijw}$. Equation (14d) computes the required number of Bell pairs for each adjacent node pair in the quantum network at window *w*. Equation (14e) defines the objective parameter- the maximum number of Bell pairs required in any adjacent node pair across the quantum network at any window *w*.

The number of binary and integer variables used in this ILP formulation is ${\mathscr {O}}(|R||V|^2|T|)$. The number of constraints used in this ILP formulation is ${\mathscr {O}}(|R||V|^2|T|)$.

## Heuristic algorithm

The optimization model described in previous section works well for small problem size only and becomes intractable for large quantum networks with a higher number of requests. A heuristic algorithm is developed and described in this section to solve this resource allocation problem for large problem size. The key issues must be taken into account while designing this heuristic are: (i) selecting a Bell generation window for each request, and (ii) selecting routing path for each request. The proposed heuristic pre-computes a set of simple shortest paths and set of eligible Bell generation window(s) for each request. A probabilistic method is used to select the Bell generation window for a request from the set of eligible window(s). The routing path is selected using a greedy approach so that the maximum number of Bell pairs required in between any adjacent Qnode pair can be minimized. The proposed heuristic is described in Algorithm 1.

The additional parameters used in Algorithm 1 are described as follows. *K* is the number of pre-computed simple shortest paths for each request *r*. $\hbox {PATH}^r$ is the set of *K* simple shortest paths for request *r* with intermediate node less than or equal to *C*. $\hbox {path}^r_K$ denotes the *K*th path of $\hbox {PATH}^r$. ${\overline{W}}$ is the set of Bell generation windows sorted in ascending order of $A_w$. ${\overline{W}}^r$ is the set of Bell generation window(s) eligible to place request *r* in *G* based on $(a^r, b^r)$. $P^r_w$ is the probability of placing request *r* in *G* in Bell generation window *w*. $G^{\prime }$ is a temporary copy of the quantum network *G* in current time-stamp. $\beta ^r_k$ is the required number of Bell pair(s) in the Qnode(s) in its routing path based on Eq. (1) for $\hbox {path}^r_K$. $\gamma ^r_k$ denotes the maximum number of Bell pairs required in any adjacent node pairs of *G* ($G^{\prime }$ after placing $\hbox {path}_K$ over *G* ($G^{\prime }$). $\Gamma$ denotes the maximum number of Bell pairs required in any adjacent Qnode pairs of *G* at any time-stamp.

**Algorithm 1:**
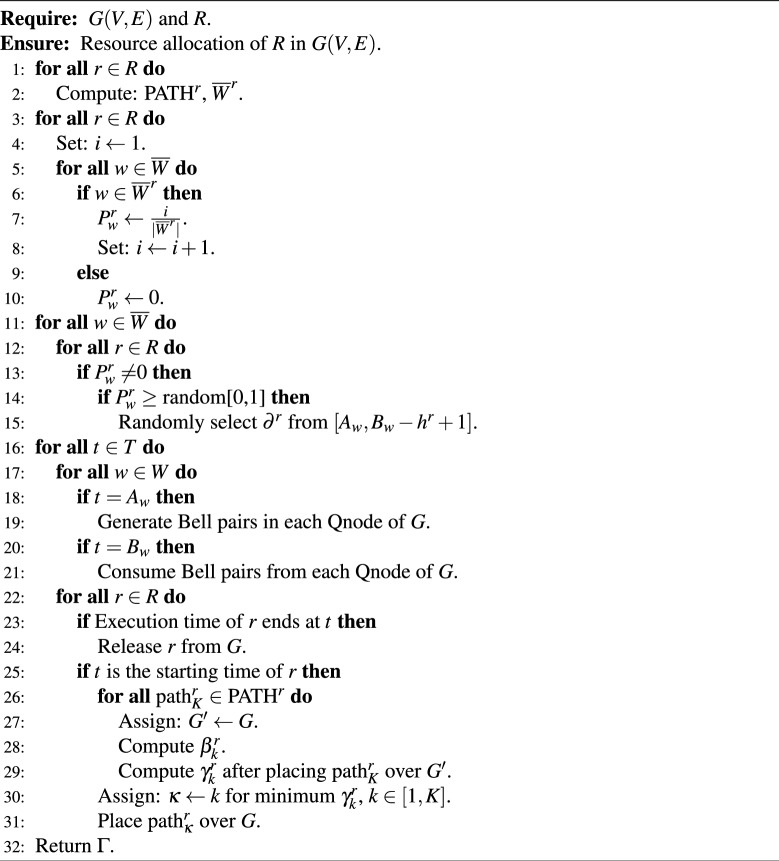
Heuristic Algorithm

Algorithm 1 begins with the request set *R* and the quantum network *G*(*V*, *E*), computes set of *K* simple shortest paths for each request $r \in R$, and stores them in $\hbox {PATH}^r$. For each request *r*, the set of Bell generation windows (${\overline{W}}$) is computed and sorted in ascending order of $A_w$. For each Bell pair generation window and each request *r*, lines 3–10 of Algorithm 1 determine $P^r_{w}$, a probability value to place request *r* in the quantum network in window *w*. An increasing probability value is assigned to the available windows for any request *r*. For example, if three Bell pair generation windows $\{w_1, w_2, w_3\}$ in ascending order of $A_w$ are available for request *r*, then values of $P^r_{w_1}$, $P^r_{w_2}$, and $P^r_{w_3}$ are $\frac{1}{3}$, $\frac{2}{3}$, and $\frac{3}{3}$=1, respectively. Note that, value of $P^r_{w}$ for any request *r* and the last available Bell pair generation window *w* in terms of time-stamp is always 1, i.e., it must be placed in the quantum network on or before the last available Bell pair generation window. If Bell pair generation window *w* is unavailable for request *r*, value of $P^r_{w}$ is set to 0.

Lines 11–15 of Algorithm 1 select a Bell pair generation window and a starting time for each request $r \in R$. A random number is generated in the range [0, 1] and compared with $P^r_{w}$ to place request *r* in window *w*. If the value of $P^r_{w}$ is greater than or equal to the generated random number, window *w* will be selected to place request *r*.

Lines 16–31 of Algorithm 1 place request set *R* in the quantum network *G* in a greedy way. At the beginning of each window, Bell pairs are generated in between each adjacent node pair and stored at the quantum memory cells (lines 18 and 19). When a Bell pair generation window ends, all the Bell pairs in the quantum network *G* are discarded (lines 20 and 21). Next, Algorithm 1 checks whether a placed request completes its execution or not; and if yes, it releases the request (lines 23 and 24). When the starting time of any request *r* is arrived, Algorithm 1 checks which path from set $\hbox {PATH}^r$ is favourable in terms of minimizing the objective function [see Eqs. (14a–14e)] based on the current condition of the quantum network. The path in which the maximum number of allocated Bell pair in adjacent node(s) is minimum is selected. In other words, the path *k* with minimum $\beta ^r_k$ value is selected for request *r* and required number of Bell pairs (gross rate) are allocated along each adjacent Qnode pair in the routing path. Algorithm 1 terminates when all requests are placed on the quantum network.

The computational complexity of Algorithm 1 is computed as follows. The complexity of computing *K*-shortest path using Yen’s Algorithm^32^ for each request is ${\mathscr {O}}(|R|K(|E|+|V|log|V|))$. The complexity to sort Bell pair generation windows is ${\mathscr {O}}(|W|log|W|)$. The worst-case complexity of line numbers 3–15 to determine eligible window(s) and select window for each request is ${\mathscr {O}}(|R||W|)$. The worst-case complexity of line numbers 16-31 to select a routing path and allocate resources on that path for each request is ${\mathscr {O}}(|T||W| + |T||R|K(|E|+|V|^2))$. Hence computational complexity of Algorithm 1 is ${\mathscr {O}}(|R|K(|E|+|V|log|V|) + |W|log|W| + |R||W| + |T||W| + |T||R|K(|E|+|V|^2))$.

## Experimental results

The performances of proposed ILP model and developed heuristic algorithm are compared in this section. The comparison of proposed approaches are performed in terms of:

(i) $\Gamma$- the maximum number of Bell pairs required in the network,
(ii) $R_{\text {LM}}$- the number of requests using routing path with length higher than the corresponding shortest path (in terms of the number of intermediate node(s)), and
(iii) fidelity in between without entanglement purification (w/o-P) and with entanglement purification (w-P) considering symmetric purification operation.

A small 9-Qnodes and a large 25-Qnodes sample grid quantum networks are considered for small scale and large scale simulations, respectively. Following parameters are considered to carried out the simulation process:

(i) |*R*|-the number of requests,
(ii) |*W*|-the number of Bell pair generation windows,
(iii) *q*-entanglement success probability, and
(iv) *B*-the net rate of incoming requests.

The simulation is performed 20 times for each set of considered parameters |*R*|, |*W*|, *q*, and *B*, and the result is obtained with 95% confidence interval. The arrival time $a^r$ of the request set is generated by poison distribution with parameters $(\frac{|T|}{4},|R|)$, and the holding time $h^r$ (in time-stamp) of each request is uniformly distributed from the range [1, 4]. The deadline of each request is randomly selected from the range $[a^r + h^r +1, |T|]$. The ILP formulation is solved using IBM ILOG CPLEX optimization studio (Version 12.8)^33^. The heuristic algorithm is evaluated using python 3.11.

**Figure 3:**
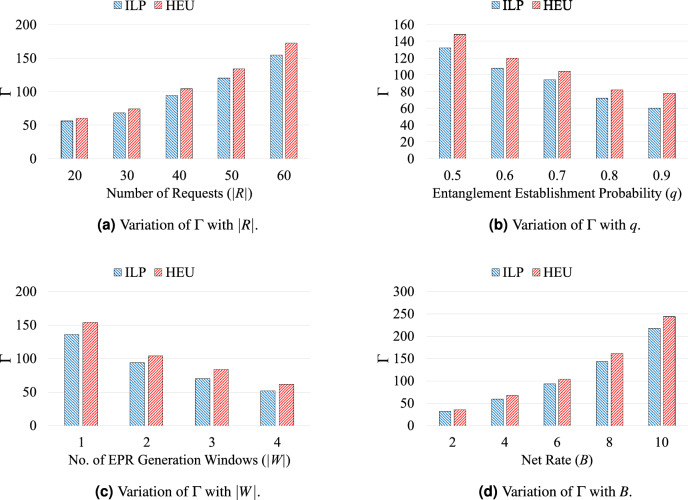
Comparison of ILP model and heuristic algorithm in terms of $\Gamma$ in different cases.

### Experimental results for small scale model

A 3$\times$3 grid quantum network topology^25,27^ with 9 Qnodes is considered to compare the performances of proposed ILP model and developed heuristic algorithm. The number of requests is varied from 20 to 60, with a step of 10, the value of entanglement success probability *q* is varied from 0.5 to 0.9, with a step of 0.1, and the value of demanded net rate *B* is varied from 2 to 10, with a step of 2. The value of total time-stamp |*T*| is set to 36, and number of Bell pair generation windows are considered as 1, 2, 3, and 4: hence respective value of quantum memory time $T_M$ is 36, 18, 12, and 9 unit time. The value of $F_{\text {ini}}$ and $F^{\prime }$ are set to 0.95^11,17^ and 0.78, respectively. The corresponding value of *C* is 4, i.e., any routing path may use at most 4 intermediate nodes.

Figure 3 reports comparison of the ILP model and heuristic algorithm in terms of $\Gamma$ and they are described as follows. Figure 3a plots the variation of $\Gamma$ with |*R*| when |*W*| is set to 2, *q* is set to 0.7, and *B* is set to 6. The value of $\Gamma$ increases with an increase in the number of requests for both ILP model and heuristic, as higher number of requests require greater amount resources (Bell pairs). The variation of $\Gamma$ with *q* is reported in Fig. 3b when |*R*| is set to 40, |*W*| is set to 2, and *B* is set to 6. It is observed that value of $\Gamma$ decreases when *q* increases in both cases, and can be described using Table 1. For a fixed value of *B*, the gross rate decreases when value of *q* is increased, and as a result the value of $\Gamma$ is decreased. Figure 3c plots the variation of $\Gamma$ with |*W*| when |*R*| is set to 40, *q* is set to 0.7, and *B* is set to 6. When the value of *R* is fixed and the value of |*W*| is increased, requests are distributed in multiple Bell pair generation windows. Hence, the number of requests to be placed in a single window is decreased and so that the value of $\Gamma$. Finally, Fig. 3d plots the variation of $\Gamma$ with *B* when |*R*| is set to 40, *q* is set to 0.7, and |*W*| is set to 2. The value of $\Gamma$ increases with an increase in the value of demanded net rate *B* for both the ILP model and heuristic.

In all these cases, ILP model provides optimal solution and the heuristic produces comparable results to the optimal one. For example, in case of Fig. 3a, the maximum deviation in the solution (value of $\Gamma$) of ILP model and heuristic is 11.6% for |*R*|=60. The same holds for other Fig. 3b–d.

**Table 2:** Variation of $R_{\text {LM}}$ with *q* and *B*.

Approach	q=0.5		q=0.6		q=0.7		q=0.8		q=0.9	
	B = 4	B = 8	B = 4	B = 8	B = 4	B = 8	B = 4	B = 8	B = 4	B = 8
ILP	0	0	0	0	2	0	5	2	7	3
HEU	0	0	0	0	1	0	2	1	4	2

Table 2 reports variation $R_{\text {LM}}$ for all considered values of *q* for *B*=4 and 8, when |*R*| is set to 60 and |*W*| is set to 1. In the considered small quantum network, it is observed that when value of *q* is too high, requests tends to use longer routing paths. This is because gross rate do not differ significantly when number of intermediate nodes in the path increases (see Table 1). The number of requests tends to use longer routing paths tends to decrease when net rate *B* increases from 4 to 8, as gross rate increases with *B*. When the value of |*W*| is increased keeping the values of other parameters fixed, no significant change is observed in the value of $R_{\text {LM}}$.

**Table 3:** Variation of fidelity with |*R*| and *B* for w/o-P and w-P schemes for symmetric purification operation.

*B*	Approach	Scheme	|*R*|				
			20	30	40	50	60
4	ILP	w/o-P	0.841	0.826	0.805	0.781	0.773
		wP	0.978	0.982	0.986	0.988	0.988
	HEU	w/o-P	0.836	0.82	0.794	0.776	0.769
		w-P	0.974	0.978	0.98	0.981	0.982
8	ILP	w/o-P	0.852	0.834	0.818	0.804	0.796
		wP	0.972	0.974	0.978	0.98	0.981
	HEU	w/o-P	0.839	0.827	0.811	0.796	0.788
		w-P	0.97	0.973	0.975	0.977	0.979

Table 3 reports the variation of fidelity for ILP and HEU approaches for w/o-P and w-P schemes for all considered values of |*R*| and *B*=4 and 8, respectively. The values of *q* and |*W*| are set to 0.9 and 1, respectively. It is observed that, with purification, the fidelity is improved by 27.81% compared to w/o-P scheme for ILP when |*R*|=60 and *B*=4. Similar results are observed for other |*R*| and *B* values as well. In all cases, heuristic approach produce close to optimal solution.

### Experimental results for large scale model

**Figure 4:**
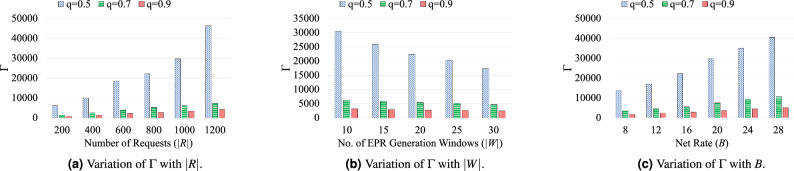
Variation of $\Gamma$ with |*R*|, |*W*|, and *B* for different values of *q*.

The performance of the heuristic algorithm is evaluated on a 5$\times$5 grid quantum network topology^25,27^ with 25 Qnodes. The number of requests is varied from 200 to 1200, with a step of 200, and value of demanded net rate *B* is varied from 8 to 28, with a step of 4. The value of total time-stamp |*T*| is set to 900, and number of Bell pair generation windows are considered as 10, 15, 20, 25, and 30, and respective value of quantum memory time $T_M$ is 90, 60, 45, 36, and 30 unit time. The value of $F_{\text {ini}}$ and $F^{\prime }$ are set to 0.95^11,17^ and 0.6, respectively. The corresponding value of *C* is 10, i.e., any routing path may use at most 10 intermediate nodes.

Figure 4 reports the performance of heuristic algorithm in terms of $\Gamma$ for *q*=0.5, 0.7, and 0.9, and are described as follows. Figure 4a plots the variation of $\Gamma$ with |*R*| when |*W*| is set to 20 and *B* is set to 16. The value of $\Gamma$ increases with increasing value of |*R*| for all considered values of *q*. The value of $\Gamma$ decreases in a rapid way when value of *q* increased from 0.5 to 0.9 for any value of |*R*|. Figure 4b plots the variation of $\Gamma$ with |*W*| when |*R*| is set to 800 and *B* is set to 16. Similar to the small scale results, value of $\Gamma$ decreases when value of |*W*| is increased from 10 to 30 for all considered values of *q*. Figure 4c plots the variation of $\Gamma$ with *B* when |*R*| is set to 800 and |*W*| is set to 20. The value of $\Gamma$ increases with an increase in the value of demanded net rate for all considered values of *q*.

**Figure 5:**
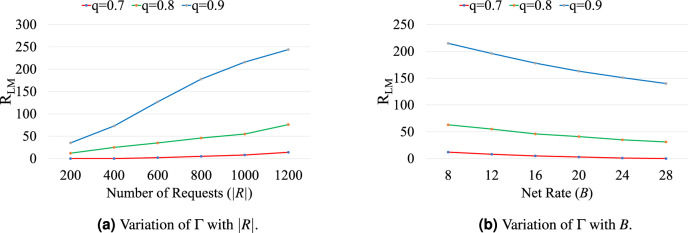
Variation of $R_{\text {LM}}$ with |*R*| and |*W*| for different values of *q*.

Figure 5 plots the variation $R_{\text {LM}}$ with |*R*| and |*W*| for *q*=0.7, 0.8, and 0.9. The variation $R_{\text {LM}}$ with |*R*| is plotted in Fig. 5a when |*W*| is set to 20, and *B* is set to 16. The value of $R_{\text {LM}}$ increases with increasing value of |*R*| for *q*=0.8 and 0.9, but remain almost same for *q*=0.7. It is observed that $R_{\text {LM}}$ is maximum for *q*=0.9 and minimum for *q*=0.7, e,g., value of $R_{\text {LM}}$ is 244, 76, and 14, for *q*=0.9, 0.8, and 0.7, respectively when |*R*|=1200. These longer paths allow load-balancing in the quantum network and minimize the value of $\Gamma$. The variation $R_{\text {LM}}$ with *B* is plotted in Fig. 5b when |*R*| is set to 1200 and |*W*| is set to 20. The value of $R_{\text {LM}}$ decreases with increasing value of *B* for *q*=0.8 and 0.9, but remain almost same for *q*=0.7. It is observed that $R_{\text {LM}}$ is maximum when *B* is minimum and minimum when *B* is maximum for any value of *q* e,g., value of $R_{\text {LM}}$ is 215, 63, and 12, when *B*=8 and 140, 31, and 0, when *B*=28 for *q*=0.9, 0.8, and 0.7, respectively.

**Table 4:** Variation of fidelity with |*R*| and *B* for w/o-P and w-P schemes for symmetric purification operation.

*B*	Scheme	|*R*|					
		200	400	600	800	1000	1200
12	w/o-P	0.786	0.752	0.743	0.731	0.716	0.698
	w-P	0.972	0.976	0.978	0.979	0.979	0.98
24	w/o-P	0.794	0.763	0.748	0.736	0.725	0.704
	w-P	0.973	0.976	0.979	0.979	0.98	0.98

Table 4 reports the variation of fidelity for w/o-P and w-P schemes for all considered values of |*R*| and *B*=12 and 24, respectively. The values of *q* and |*W*| are set to 0.9 and 20, respectively. With purification, the fidelity is improved by 40.4% compared to w/o-P scheme when |*R*|=1200 and *B*=12. Similar results are observed for other |*R*| and *B* values as well.

From the obtained experimental results discussed above, it can be concluded that the value of maximum utilized Bell pairs in any adjacent node pair $\Gamma$ in a quantum network increases when (i) the number of requests |*R*| to be placed increases, (ii) the value of entanglement establishment probability *q* decreases, (iii) the number of Bell pair generation window |*W*| increases, and (iv) the net rate demanded by requests *B* increases. It is noted that the requests tend to use longer routing paths when value of entanglement establishment probability increases to allow load-balancing in the quantum network. The fidelity of Bell pairs increases in case of end-to-end entanglement distribution when entanglement purification operation is performed.

## Conclusion

In this paper, we considered the offline resource allocation problem in quantum networks and proposed a routing and end-to-end entanglement distribution model considering factors like entanglement distribution probability, quantum memory, and fidelity. We proposed an optimization model and formulated it as an integer linear programming model to obtain the optimal solution of this problem in small quantum networks. We developed a heuristic algorithm to solve this problem for large problem size as the ILP formulation is tractable for small problem sizes only. The performance of proposed ILP and heuristic are compared considering extensive simulation scenarios by varying the number of requests, value of entanglement establishment probability, number of Bell pair generation windows, and demanded net rate by the requests. It is observed that the developed heuristic provided near to the optimal solutions obtained by the ILP formulation. Experimental results ensure that the value of maximum utilized Bell pairs in a quantum network increases when the value of entanglement establishment probability decreases and the number of Bell pair generation windows increases along with the number of requests and demanded net rate. In this work, we considered homogeneous quantum repeater chain, i.e., all Qnodes had the same entanglement establishment probability. Consideration of stochastic analysis with queuing theory for online traffic model in quantum networks is left as the part of our future work.

## Data Availability

Data is provided within the manuscript or supplementary information. Raw data in Excel spreadsheets. Also source code for ILP and Heuristic.
